# Genome editing for grass improvement and future agriculture

**DOI:** 10.1093/hr/uhae293

**Published:** 2024-10-15

**Authors:** Muhammad Bilal, Jie Geng, Lin Chen, Pedro García-Caparros, Tao Hu

**Affiliations:** State Key Laboratory of Herbage Improvement and Grassland Agro-ecosystems, College of Pastoral Agriculture Science and Technology, Lanzhou University, Lanzhou 730020, China; State Key Laboratory of Herbage Improvement and Grassland Agro-ecosystems, College of Pastoral Agriculture Science and Technology, Lanzhou University, Lanzhou 730020, China; State Key Laboratory of Hybrid Rice, College of Life Sciences, Wuhan University, Wuhan 430072, China; Agronomy Department of Superior School Engineering, University of Almería, Almeria, Spain; State Key Laboratory of Herbage Improvement and Grassland Agro-ecosystems, College of Pastoral Agriculture Science and Technology, Lanzhou University, Lanzhou 730020, China

## Abstract

Grasses, including turf and forage, cover most of the earth’s surface; predominantly important for land, water, livestock feed, soil, and water conservation, as well as carbon sequestration. Improved production and quality of grasses by modern molecular breeding is gaining more research attention. Recent advances in genome-editing technologies are helping to revolutionize plant breeding and also offering smart and efficient acceleration on grass improvement. Here, we reviewed all recent researches using (CRISPR)/CRISPR-associated protein (Cas)-mediated genome editing tools to enhance the growth and quality of forage and turf grasses. Furthermore, we highlighted emerging approaches aimed at advancing grass breeding program. We assessed the CRISPR-Cas effectiveness, discussed the challenges associated with its application, and explored future perspectives primarily focusing on turf and forage grasses. Despite the promising potential of genome editing in grasses, its current efficiency remains limited due to several bottlenecks, such as the absence of comprehensive reference genomes, the lack of efficient gene delivery tools, unavailability of suitable vector and delivery for grass species, high polyploidization, and multiple homoeoalleles, etc. Despite these challenges, the CRISPR-Cas system holds great potential to fully harness its benefits in grass breeding and genetics, aiming to improve and sustain the quantity and quality of turf and forage grasses.

## Introduction

Grasses are defined as wild forage or cultivated herbaceous plants [1], which dominate the grasslands and cover ~41% of the total land area, extending across both terrestrial and underwater surfaces [2, 3]. Grasses have important connection with human history and evolution, as they originated in the Late Cretaceous (100 Ma) and established on Earth in the pre-civilization period. Since the emergence of agricultural societies, the cultivation of grasses as crops has persisted and continues to be integral to human subsistence and agriculture today [4, 5]. However, as the world population increases and available resources decrease, it is essential to develop new crop and grass varieties through advanced breeding techniques that can meet present and future food and feed demands. Previous breeding techniques, such as conventional selection and crossbreeding, are laborious and time-consuming approaches for developing new varieties [6]. Therefore, in this context, we should develop or optimize new breeding techniques to produce high-yielding, high-quality, and highly resistant varieties, especially for turf and forage grasses.

Genome editing represents a successful and forward-looking approach in molecular breeding, offering the potential to revolutionize agricultural and biological research. Among the various genome editing techniques, the advent of clustered regularly interspaced short palindromic repeat (CRISPR)-CRISPR-associated protein (Cas) has been particularly transformative. As one of the most prominent genome editing tools, CRISPR-Cas has ushered in a new era of modern plant breeding [7]. CRISPR-mediated tools, such as base editors and prime editors (PEs), have significantly expanded gene editing in plants; however, their application is primarily limited to small nucleotide changes, e.g., single-nucleotide polymorphisms (SNPs), point mutations, and small insertions or deletions (indels). Recent advancements in genome editing tools have enabled the insertion of large DNA sequences with greater precision and efficiency. Techniques such as programmable addition via site-specific targeting elements (PASTE) [8] and prime editing-mediated Recombination Of Opportune Targets (PrimeRoot) have been developed to facilitate these complex genetic modifications [9]. Besides, targeted mutagenesis that do not require DNA double-strand breaks (DSBs) are gaining attention, as traditional approaches like homology-directed repair (HDR) and nonhomologous end joining (NHEJ) frequently result in unwanted defects. The efficiency of genome editing for precise genetic modifications in plants is also significantly influenced by the transformation techniques. These techniques tend to be more complex in plant cells compared to animal cells, largely due to the unique structural and physiological characteristics of plant cells [10]. A fast and efficient method for obtaining transgenic plants without undergoing tissue culturing is the cut-dib-budding (CDB) delivery system [11–15]. Despite such advancements, the application of genome editing in turf and forage grasses remains a largely under-explored domain. This is primarily due to the limited availability of ideal *in vitro* regeneration methods, efficient delivery systems, less information about the desired target loci, and suitable Cas protein for successful gene editing. As a result, contemporary grass genome editing lags behind the progress achieved in economically important crops (wheat, maize, rice, cotton, and sugarcane, etc.).

With the rapid development of genome editing technologies, especially the advent of CRISPR-Cas9, the research on the use of genome editing to improve turf and forage grass varieties for stress resistance and yield enhancement have gradually increased since 2010 [16–18]. Thus, we tried to provide an overview of the current status of genome editing studies on turf and forage grasses, aiming to provide a comprehensive guide for the application of CRISPR-Cas technology in grass breeding. This includes a detailed examination of advanced genetic knowledge and specific challenges associated with leguminosae and poaceas grasses. Furthermore, we discuss the ongoing challenges and future perspectives of genome editing for the improvement of forage and turf grasses.

## Overview of genome editing

Genome editing is a molecular manipulation technique that uses sequence-specific nucleases (SSNs) to cut DNA and create DSBs at specific sites in the genome. These breaks activate endogenous repair mechanisms in the cells through NHEJ or HDR to achieve precise modifications for the targeted genome, including gene knockout, exogenous fragment insertion or exchange of specific DNA fragment, etc. [19, 20]. Genome editing technologies have advanced rapidly over the past decade in the design and applications of various targeting nucleases. In general, genome editing technology has undergone three iterations. After the first discovery of zinc finger protein in 1984, it was artificially modified and fused with *Fok*I nucleic acid endonuclease to develop the first-generation gene editor-zinc finger nucleases (ZFNs) [21]. One of the earliest applications of ZFNs in plants was reported in 2009, when researchers used ZFNs to generate targeted mutations in the maize genome by cleaving at the *IPK1* locus [22]. Concurrently, Townsend *et al*. [23] reported the induction of herbicide resistance mutations in tobacco *ALS* genes through ZFN-mediated gene targeting. Following this, the second-generation gene editor, the transcription activator-like effector nucleases (TALENs), started being used in plants immediately after its discovery to create targeted genome modifications [24]. The earliest applications of TALENs in plants were in the model plant *Arabidopsis thaliana* [25], as well as in non-model plants such as rice [26], tobacco [27, 28], barley [29], and maize [30], among others. These genome editing technologies are similar but more efficient to ZFNs and consist of two components: the TALE region, which recognizes and binds to the DNA, and the *Fok*I nucleic acid endonuclease region, which cuts the DNA [28]. Despite their effectiveness, ZFNs and TALENs, which use protein–DNA binding to target sites for targeted cleavage, have not been widely used due to their complexity, low efficiency, and narrow range of applicable species [31]. While these SSNs have proven to be very effective for plant genome editing, the intricate protein assembly has constrained their practical application.

**Figure 1:**
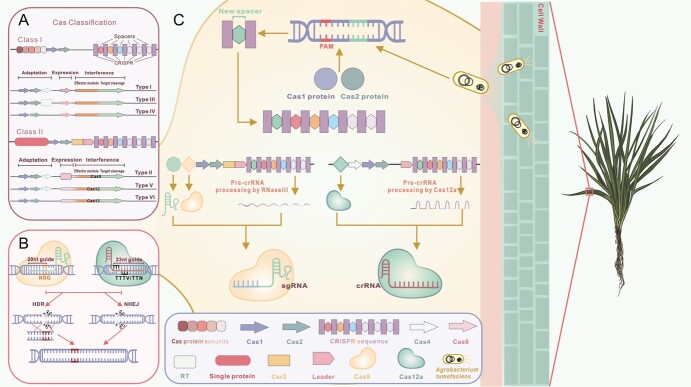
CRISPR-Cas system classification, structures, and mechanisms. CRISPR-Cas system classification (A). The CRISPR-Cas system can be divided into two classes depending on the number of Cas proteins that form effector modules. Class I uses effector protein complexes composed of multiple Cas proteins that form a crRNA-binding complex. Class II has a single multidomain crRNA-binding protein. Each class contains three types. The scheme shows the typical relationships between the genetic, structural and functional organizations of the six types of CRISPR/Cas systems. CRISPR/Cas9 system and CRISPR/Cas12a system’s structures (C) and mechanisms (B). The immune defence presented on the upper contains three steps: spacer acquisition (C), crRNA biogenesis (C), and target interference (B).

The CRISPR-Cas system, which was firstly identified in the *E. coli* genome in 1987 [32], is a complex adaptive immune system prevalent in bacteria and archaea [33]. This system can recognize exogenous invasive nucleic acid sequences and specifically degrade them to provide protection against extracellular entities such as viruses [34]. In this system, bacteria have developed a clever way to detect and destroy viral DNA to defend themselves. When a virus attacks, the bacterium recognizes snippets of the virus’s genetic code and produces small RNA molecules with matching sequences. These RNA molecules bind to a Cas9 protein. Remarkably, Cas9 acts like molecular scissors that can cut DNA. When the RNA guides Cas9 to the matching viral sequence, it slices the DNA, crippling the virus. Scientists have adapted this bacterial defense mechanism into a powerful groundbreaking tool known as CRISPR genome editing. This technique enables precise modification of genetic material like editing a document on a screen. The process has three basic steps: targeting, cutting, and repairing. Researchers design RNA molecules to target a specific DNA sequence. These “guide RNAs” shepherd the Cas9 protein to its target, like handing scissors to someone and pointing to a spot in a book. Only when the RNA and Cas9 encounter the right sequence does the protein spring into action, snipping the double-stranded DNA. The cell’s repair machinery then patches the break, allowing scientists to insert new genetic code at that location. CRISPR holds significant potential for correcting genetic defects and treating a broad spectrum of diseases [35, 36]. To provide a comprehensive understanding of the CRISPR-Cas system, Figure 1A–1C illustrates its classification, mechanism and structure. In 2013, the CRISPR-Cas9 system was initially introduced to plants to evaluate its effectiveness in genome editing. Simultaneously, *A. thaliana* [37] and *Nicotiana benthamiana* [38] were selected as model organisms to assess the feasibility and efficacy of mutagenesis in specific genomic regions [39]. Additionally, the system was applied to wheat and rice to investigate sequence-specific genome modifications, demonstrating the versatility and potential of genome editing technologies across diverse plant species [40].

Based on the Cas gene core element sequences, CRISPR-Cas systems have been divided into 2 classes, 6 types, and 33 subtypes: Class I CRISPR-Cas systems utilize multiple Cas protein complexes for target site cleavage, including types I, III, and IV, while Class II CRISPR-Cas systems can employ a single Cas protein in complex with CRISPR RNAs (crRNAs) for interference, including types II, V, and VI [41]. The CRISPR-Cas9 system as a class 2 type II CRISPR system was successfully implemented for the first time in various cell types and organisms in 2012–2013. Jiang *et al*. [42] applied nucleotide complementary pairing to combine at genomic target sites for cleavage. After 2 years of CRISPR’s discovery, Class 2 of CRISPR-Cas came into existence; Cas12a protein (initially known as Cpf1) is an upgrade in the CRISPR-Cas system, which is smaller in size than Cas9 that can help in terms of delivery to the plant cells [43]. CRISPR-Cas12a system was used for the first time in rice and tobacco; and these studies reported that the alteration in the sequence is heritable [44, 45]. Although the CRISPR-Cas12 system is a more recent advancement than the CRISPR-Cas9 system, it has some specificities that enhance its efficiency compared to the CRISPR-Cas9 system. However, the CRISPR-Cas9 system is still believed to be significantly smarter and more efficient [46]. The allegiance of the CRISPR-Cas9 system to gene editing made it more efficient, popular and most mature gene editing system by dramatically impacting the life science field.

**Figure 2:**
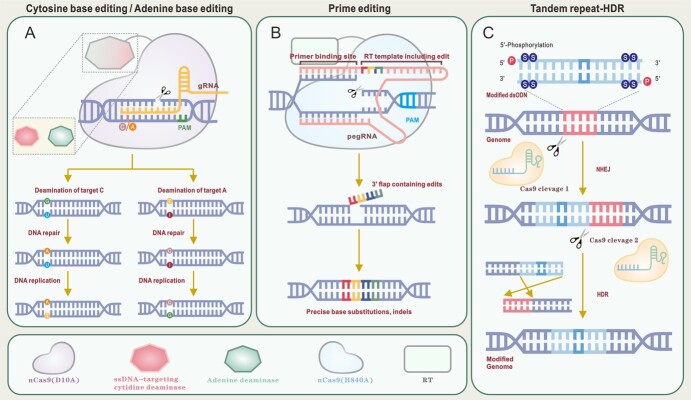
Graphical representation of various genome editing techniques. Base Editing (Cytosine/Adenine Base Editing): Base editors enable precise nucleotide substitutions by combining a deaminase enzyme (cytidine deaminase for C-to-T substitution or adenine deaminase for A-to-G substitution) with a Cas9 nickase (nCas9; D10A) (A); prime editing uses a Cas9 nickase mutant (nCas9(H840A)) and a prime editing guide RNA (pegRNA). One end of the pegRNA functions as a reverse transcription primer binding site (PBS), while the opposite end acts as a reverse transcription template that includes the intended edit, allowing it to bind to reverse transcriptase (RT). The H840A mutant of nCas9 selectively nicks only the DNA strand of the target containing the protospacer adjacent motif (PAM) (B); Tandem repeat homology-directed repair (TR-HDR) is designed to facilitate the insertion and replacement of large DNA fragments. This technique involves the artificial creation of tandem repeats at the target site following the insertion of the modified fragment, thereby enhancing the efficiency and accuracy of homology-directed repair (C).

In the pursuit of enhanced editing efficiency and precision, CRISPR-Cas-based genome editing techniques in plants continue to evolve and innovate [47]. In addition to these advancements, base editing (BE) technology has emerged as a significant breakthrough, enabling the direct conversion of nucleotide bases within a DNA sequence without requiring DSBs [48]. The discovery of base editors in 2016 marked a significant advancement in genetic engineering, and the technology continues to evolve with each successive generation. BE is an advanced genome editing technology that does not rely on HDR but is based on single precise base substitution or transversion (C to T, A to G, T to C, or G to A), including cytosine base editor (CBE) and adenine base editor (ABE, Fig. 2A) [49, 50]. CBEs utilize a single-stranded DNA-targeting (ssDNA) cytosine deaminase, such as rat APOBEC1 (rAPOBEC1) or *Petromyzon marinus* cytidine deaminase 1 (PmCDA1), linked to an uracil DNA glycosylase inhibitor (UGI). This makes a complex with nickase Cas9 (nCas9, D10A) and facilitates the precise conversion of cytosine–guanine (C•G) base pairs to thymine-adenine (T•A) base pairs through the formation of an uracil intermediate, resulting in an uracil–guanine (U•G) mismatch [51, 52]. Bottero et al. [53] used CBEs to edit ALS genes and efficiently get single BE (C to T base conversion) in alfalfa. ABEs mainly consist of three components: Cas9 nickase (nCas9), an engineered adenine deaminase enzyme and a guide RNA (gRNA). This nCas9-gRNA complex locates and binds to the target sequence. Adenine deaminase catalyzes the conversion of adenine (A) to inosine (I), thereby enabling A-to-G transitions within a narrow editing window [54, 55]. It is possible to use base editors in grass genome editing; however, genome editing in turf and forage grasses using base editors is still in its early stage [53], while these BE tools are being widely used in various cultivated crops [56–60]. In 2021, Wei *et al*. introduced a newly optimized highly efficient adenine base editing system (ABE8e) that nearly performed up to 100% editing efficiency and was also effective in the homozygous base substitution [61]. It has become fruitful and is being implemented in modern molecular plant breeding. Unfortunately, BE is limited by its inability to facilitate inter-base reversal or editing of small fragments. In 2019, the introduction of PE represented a significant advancement in genome precision editing technology, elevating the field to new levels of accuracy and versatility [62]. Prime editing is an advanced genetic engineering technology designed to install precise small insertions and deletions (indels), as well as various types of single or multiple base substitutions, including both transitions and transversions, and their combinations. Notably, this technique achieves these modifications without the necessity of DSBs or donor repair templates (DRTs) (Fig. 2B) [63]. PEs employ nCas9 H840A fused to a reverse transcriptase derived from Moloney murine leukemia virus (M-MLV). A prime editing guide RNA (pegRNA) comprises a spacer at its 5′ end, a reverse transcription template, and an additional primer binding site at its 3′ end. The nCas9 (H840A) induces a nick in the nontarget strand. Subsequently, the primer binding site sequence forms a hybrid with the 5′ end of the nick, starting the process of reverse transcription. This process provides a 3′ flap containing the desired modifications, which can be integrated into the DNA repair. By searching and replacing target sites, PE can achieve any transformations between four bases and accurately insert or remove small fragments, overcoming the low efficiency of CRISPR-Cas9 system-mediated HDR and the inability of BE to achieve base reversal [64]. A new strategy, tandem repeat homology-directed repair (TR-HDR) was devised to achieve efficient sequence insertion and replacement in the rice genome; where TR-HDR showed better results with 6.1% efficiency up to 130-base pair sequence modification than CRISPR-Cas9 system-mediated HDR. TR-HDR leverages tandem repeats at the target site, chemically modified donor DNA, and HDR to efficiently modify genes using CRISPR-Cas9. Tandem repeats help recruit donor DNA, while chemical modification and the HDR process enable accurate and highly efficient gene replacement (Fig. 2C) [65]. Each gene editing technique has been developed with significant advantages over conventional breeding methods [63] and their increasing application provides new pathways for genome editing in grasses.

## Genome editing in grasses

**Figure 3:**
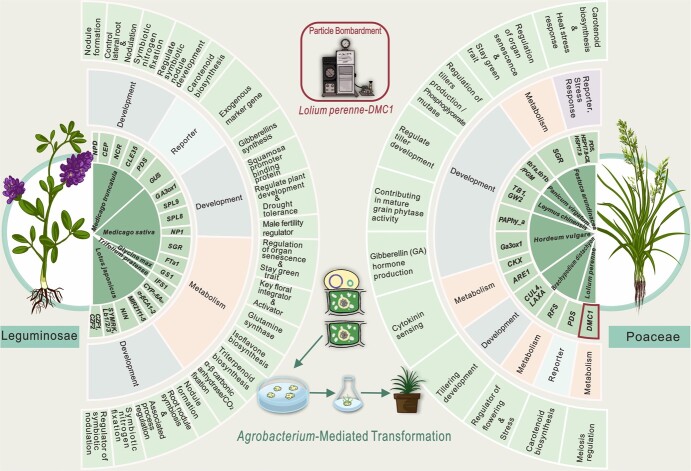
Graphical summary of CRISPR/Cas9-mediated genome editing in turf and forage grasses. From the inner circle to the outer circle are: demonstrated Species, targeted gene names, targeted gene classification, and gene functions. Most of edited genes were delivered by *Agrobacterium tumefaciens*-mediated plant transformation. Only *LpDMC1* (circled in red) was transformed by particle bombardment-mediated transformation.

In recent years, researchers have successfully sequenced the genomes of several grass species, including ryegrass (*Lolium perenne* L*.*) [66], orchardgrass (*Dactylis glomerata* L.) [67], and alfalfa (*Medicago sativa* L.) [68], among others. Despite these advancements, identifying and analyzing genes that influences critical agronomic traits in many grasses remain challenging. This difficulty arises from the inherent complexities of these species, such as self-incompatibility, cross-pollination, and polyploid inheritance [69]. To date, the majority of grasses are stuck in the stage of hybrid breeding, lagging behind other major crops in terms of genetic advancement. Consequently, grass breeding has a broad development space and great significance [70]. After the initial successful implementation of genome editing in grasses via CRISPR-Cas9 technology in 2013 [38, 40], an increasing number of researchers striving to establish efficient genetic transformation systems and genome editing systems for a variety of turf and forage grasses, providing valuable research tools for creating new germplasm with excellent quality. To date, more than 11 grass species have been successfully edited by CRISPR-Cas9/Cas12a technologies (Fig. 3, Table S1).

## Genome editing in Leguminosae grasses

In forage legumes, most genetic studies are focused on targets affecting the legume/rhizobia symbiosis, followed by targets affecting biosynthesis and secondary metabolic processes. *Medicago truncatula* and *Lotus japonicus* are used as the model organisms commonly engaged in symbiotic nitrogen fixation studies. In contrast, *M. sativa* recognized as the most productive perennial legume globally and has been the focus of various genome editing experiments aimed at enhancing its traits and improving its agricultural performance [71]. As far as *M. truncatula* was concerned, Michno *et al*. [72] developed an online web tool to efficiently identify CRISPR-Cas9 target sites. The researchers used this webserver to design a soybean codon-optimized CRISPR-Cas9 platform for gene editing of the soybean *Glutamine Synthase* (*GS1*) gene. They subsequently tested this system for its ability to cause targeted mutagenesis in the *β*-glucuronidase (*GUS*) reporter gene in *M. truncatula*. Both soybean and *M. truncatula* were transformed by hairy root transformation, respectively. Molecular studies revealed a series of mutations in the target genes in the hairy root-transformed soybean and *M. truncatula* cells. Additionally, a study of *M. truncatula* demonstrated the phenotypic changes in nodule formation and development through mutating *NPD* genes via CRISPR-Cas9 technology [73]. Zhu *et al*. [74] studied *MtCEP* gene function in lateral root inhibition and promoting nodulation using an optimized CRISPR-Cas9 toolkit and reported that the mutants did not show any significant results. Nevertheless, *MtCEP_1_,_2_,* and *_12_* were verified to be superfluous in promoting nodulation and lateral root inhibition. Güngör *et al*. [75] used the CRISPR-Cas9 system to perform targeted mutagenesis in *NCR* genes using the *Agrobacterium*-mediated transformation. They produced mutants with edited *NCR* genes via the CRISPR-Cas9 system, and gene editing helped to analyze that there were four peptides, NCR068, NCR089, NCR128, and NCR161, which were dispensable for symbiotic nitrogen fixation. In another study of *M. truncatula*, it was discovered that the *MtCLE35* gene, after being edited with the CRISPR-Cas9 technology, produced an increased number of nodules when nitrate was present compared to wild-type plants. Nevertheless, other related genes decreased in their expression level [76].

There are relatively few reports on the study of *Lotus japonicus* (Regel) K. Larsen. Wang et al., edited the *LjSYMRK* gene in *L. japonicus*, which is associated with symbiotic nitrogen fixation [77]. This modification was achieved by *Agrobacterium*-mediated stable or hairy root transformation using the CRISPR-Cas9 system. The SYMRK-sgRNA was constructed based on the selection target in *LjSYMRK* and obtained ~35% mutagenic efficiency in 20 T0 transgenic plants. Among these, two T0 mutants successfully obtained biallelic homozygous mutations with a 2-bp deletion. Furthermore, two sgRNAs were further designed to target three particular leghemoglobin loci (*LjLb1*, *LjLb2*, and *LjLb3*), and multiple knockouts of *LjLb1*, *LjLb2*, and *LjLb3* were successfully achieved, resulting in the production of white nodules, thereby demonstrating that the CRISPR-Cas9 system is capable of effective editing of one or more genes in *L. japonicus.* Based on the project study, Wang *et al*. successfully created *leghemoglobins* (*Lbs*) single, double, and triple mutants using the CRISPR-Cas9 system. Their findings confirmed the crucial involvement of *Lbs* in symbiotic nitrogen fixation in *Lotus japonicus*. Notably, the knockout of *Lbs* led to the development of premature nodules [78]. In the same way, working with nodule-enhanced carbonic anhydrase genes, the knockout mutants of *LjβCA1* or both *LjαCA2* and its homolog generated by CRISPR-Cas9 technology did not result in phenotypic differences compared to wild-type plants indicating that *LjβCA1* or *LjαCA2* genes were not essential for nitrogen fixation under normal symbiotic circumstances [79]. Cai *et al*. [80] used the CRISPR-Cas9 system to interrupt the functioning of both *LjCZF1* and *LjCZF2* genes, which encode the Lotus Histidine Kinase 1(LHK1)-interacting proteins responsible for cytokinin receptor activity via hairy root transformation. The *LjCZF1* and *LjCZF1*-*LjCZF2* mutants exhibited a reduced number of nodules compared to the empty vector (EV) transgenic lines, confirming that *LjCZF1* has a beneficial effect in controlling rhizobial infection and nodule organogenesis in *L. japonicus*. Suzuki *et al*. [81] demonstrated that the *CYP716A51* was necessary for triterpenoid biosynthesis in *L. japonicus* by knocking out them using the CRISPR-Cas9 system and performing gene function deletion analysis. Akamatsu *et al*. [82] edited the promoter region of the transcription factor NIN using CRISPR-Cas9 technology to generate a *CYC-RE* deletion mutant. The loss of the *CYC-RE* region resulted in a significant decrease in infection, indicating that *CYCLOPS* is accountable for NIN expression in this mechanism. The CRISPR-Cas9-mediated knockouts of *MIR2111–5* containing dual gRNA in *L. japonicus* reduced the nodule number. Moreover, using different types of scions in grafting experiments revealed a clear correlation between the type of rootstocks and nodule number, highlighting the influence of rootstock selection on nodule formation [83].

Alfalfa (*M. sativa* L.) is an excellent forage legume, which is known as the “king of forage grasses” due to its abundant protein and adaptable characteristics [84]. However, its complex genetic characteristics have caused a relative delay in studies on gene mining and genetic improvement of essential traits. Alfalfa is closely related to *M. truncatula* and has a high genetic similarity with other legume forage grasses. Completing the whole genome sequence of *M. truncatula* has become an exemplary model for studying genetics and functional genomics of legumes, providing opportunities for expanding the genomic toolbox of alfalfa [85]. Meng *et al*. [86] established an efficient CRISPR-Cas9 genome editing system for *M. truncatula* through *Agrobacterium*-mediated transformation using a native Medicago U6 promoter combined with a codon-optimized Cas9 protein. Their system successfully performed a targeted knockout of the endogenous gene *MtPDS* in alfalfa, obtaining 10.35% pure T0 generation knockout plants with albino phenotype. This study provided new research tools for gene editing in alfalfa. On the same hand, Zhang *et al*. [87], to target the *MtPDS* gene, achieved an amalgamation of the CRISPR-Cas9 system and the hairy root system where the seedlings displayed albino phenotypes regenerated from the homozygous/biallelic hairy root mutation lines of *MtPDS*. In the first round of hairy root culture, they got 5% fewer mutations than the previous study reported [86]. However, in the second round, using branch roots, they obtained up to 75% of the homozygous lines. Additionally, Curtin presented a protocol demonstrating the successful generation of mutant plants in *M. truncatula* and *M. sativa* using reagents constructed from two genome engineering platforms [88]. Gao *et al*. successfully edited the *squamosa promoter binding protein-like 9* (*SPL9*) gene in alfalfa by using CRISPR-Cas9 system, which provided technical support and new opportunities for application in future alfalfa breeding [71]. However, further exploration is needed on how to improve the efficiency of gene editing. A subsequent system was entrenched based on efficient multiple genome editing techniques to outcross tetraploid alfalfa. The application of this system revealed that mutation efficiency was increased at significant levels by Arabidopsis UBQ10 promoter-driven Cas9, with 95% in Arabidopsis and 70% in *M. truncatula* [89]. In the same year, Chen *et al*. [68] completed deciphering the autotetraploid cultivated alfalfa genome for the first time and devised a very effective genome editing protocol using the CRISPR-Cas9 system. This system has not only successfully created several new materials of multi-leaf alfalfa, but it is also vital that the application of this technology does not require the introduction of exogenous genes and transgenic process, just only the acquisition of its mutants, which will significantly accelerate the speed of the traditional breeding methods. For the high genome editing efficiency in *M. sativa*, Wolabu *et al*. [90] optimized the multiplex gRNA-CRISPR-Cas9 system and got a 75% mutation in targeting the *MsSGR* gene. Unlike the study conducted by Michno *et al*. [72], Bottero *et al*. [91] generated two genetic manipulation events using CRISPR-Cas9 with the pBI121 binary vector, which contains the *GUS* gene and observed an average of 55% inactivation of the *GUS* gene (efficiency of CRISPR-Cas9 genome editing) in alfalfa. Singer *et al*., using CRISPR-Cas9 in the alfalfa *SPL8* gene, produced distinct phenotypic changes in *MsSPL8* mutants. These changes included reductions in flowering time, leaf size, internodal length, plant height, shoot and root biomass, and root length. Additionally, the mutants exhibited an increased degree in drought tolerance [92]. Zheng *et al*. [93] obtained prostrate and semi-dwarf alfalfa by CRISPR-Cas9-based gene editing in the *MsGA3ox1* gene where the mutants showed a notable reduction in plant height and internodal length as well as increased high crude protein and leaf/stem ratio. Ye *et al*. [94] cracked the problem of the alfalfa seed industry and made a breakthrough in the research of using gene editing to create genic male sterile lines. They used efficient gene editing tools inspired to target *MsNP1* sites in alfalfa to obtain mutant materials efficiently, providing a vital material resource and methodological basis for utilizing hybrid advantage in alfalfa [74]. Their gene editing efficiency targeting *MsNP1* achieved 96.9% in 62 of 64 transgenic lines. In a recent study, Wolabu *et al*. [17] used the multiplex CRISPR-Cas9 system and *Agrobacterium*-mediated transformation to target the *MsFTa1* gene, which relates to the flowering of alfalfa. They observed that 22 out of 96 putative mutant lines got retarded flowering time and even 6 independent Msfta1 lines had mutations in their all four copies of *MsFTa1*. The delay in flowering time in alfalfa mutants resulted in increased plant height and biomass.

In addition, there is a successful study to knock out the *isoflavone synthase* (*IFS1*) gene, a crucial enzyme involved in the synthesis of isoflavone in red clover (*Trifolium pratense*), using the CRISPR-Cas9 system. A hemizygous plant with a 9-bp deletion in the *IFS1* gene was obtained, and then pure homozygous mutant plants were intercrossed with them. The inoculation of wild-type and editing-mutant plants with rhizobia did not result in any notable alteration, indicating that isoflavones did not play a significant role in the nodulation process [95]. The aforementioned studies highlight the significant contributions of CRISPR-Cas9 technology for functional genomics research and offer novel approaches for advancing genetics and molecular breeding in forage legumes with complex genomes.

## Genome editing in Poaceae grasses

To date, considerable successful applications of CRISPR-Cas9/Cas12a technology have been reported in *Brachypodium distachyon*, switchgrass (*Panicum virgatum* L*.*), perennial ryegrass (*L. perenne* L*.*), tall fescue (*Festuca arundinacea* Schreb.), barle*y* (*Hordeum vulgare* L*.*), *Echinochloa frumentacea* (Roxb.) Link, sheepgrass (*Leymus chinensis* (Trin.) Tzvelev), and Zoysia grass (*Zoysia japonica*).

*B. distachyon* is the first crop within the Pooideae sub-family to have its whole genome sequenced [96], being an ideal model plant for functional genomics research in *Poaceae grasses* and effective implementation of CRISPR-Cas9 technology [97]. Hus *et al*. developed a comprehensive procedure for precise modification of the genome of *B. distachyon* and its allotetraploid derivative *B. hybridum*. They successfully targeted five distinct genes (*PDS*, *FLA*, *PME*, *CDKG1*, and *CDKG2*) in *B. distachyon*, and two of them (*CDKG1* and *CDKG2*) in *B. hybridum* [98]. A recent study on *B. distachyon* demonstrates that the *BdRFS* gene is related to stress inducive metabolism and the regulation of flowering. This study explained that the overexpression of the *BdRFS* gene in *B. distachyon* delayed its flowering and boosted drought resilience and biomass acquisition. However, using the CRISPR-Cas9 system to knock out the *BdRFS* gene produced the opposite trend; since CRISPR mutants showed hypersensitivity to water and reduced pigmentation and thickness of mature leaves and early flowering with decreased biomass [99]. Furthermore, Li *et al*. [100] suggested CRISPR-Cas9 technology to disable the *FRUITFULL* genes (*BdFUL3* and *BdFUL4*) in *B. distachyon*. Still, no phenotypic changes were observed, probably because these two genes have functional redundancy with other genes. The generation of null alleles in two *NBCL* (*NOOT-BOP-COCH-LIKE* genes with an essential role in vegetative and reproductive development) paralogs *BdUNICULME4* (*CUL4*) and *BdLAXATUM-A* (*LAXA*) via CRISPR-Cas9 editing approaches resulted in dwarf phenotypes linked to the cell inhibition in stems [101].

As a member of the Poaceae family, switchgrass is a perennial C_4_ grass species notable for its ability to thrive under different soil conditions, even tolerating acidic soils and defoliation in grazing [102, 103]. For the study of switchgrass, the tiller-related genes *tb1a* and *tb1b* and the phosphoglycerate mutase *PGM* gene were effectively altered using a Cas9/sgRNA complex through *Agrobacterium*-mediated stable transformation of embryogenic calli [104]. Mutation frequencies of 95.5% (*tb1a*) and 11% (*tb1b*) were obtained in T0 generation using a single construct that had two gRNAs targeting distinct areas of two genes, respectively. Meanwhile, the targeted mutagenesis of the *PGM* gene had an editing efficiency of 13.7% in mutants with a CRISPR-Cas9 construct that included a solitary single sgRNA [104]. Two years later, Liu *et al*. [105] reported in their new study that they successfully generated nonchimeric *Pvtb1a* and *Pvtb1b* mutants by nodal culture from chimeric T0 mutants. Transcriptome analysis revealed that 831 genes exhibited differential expression in the *Pvtb1a-Pvtb1b* double knockout mutants. This indicates that *Pvtb1* genes play a crucial role as a negative regulator in the production of tillers in switchgrass. Furthermore, it provides an opportunity to clarify further the molecular pathways that control tillering in switchgrass.

Ryegrass (*Lolium* spp.) is known for its comprehensive cultivation in temperate climate regions due to its notable attributes as a perennial forage grass. Its high digestibility and grazing tolerance enhance its suitability for use in sports grounds, lawns, and parks [106, 107]. Reports on ryegrass are relatively sparse. In one report, Zhang *et al*. [108] introduced mutations in a meiotic recombinase gene *LpDMC1* in Italian ryegrass (*Lolium perenne ssp. Multiflorum*) and perennial ryegrass using the CRISPR-Cas9 system and obtained 8 T0 heterozygous mutants. *Lpdmc1* mutants exhibited complete male sterile and significantly disrupted meiosis, suggesting that *DMC1* is a highly conserved protein that functions critically in meiosis. In another study, Kumar *et al*. [107] used the Cas9-sgRNA transcript system and edited the *PDS* genes with 29% efficiency of bi-allelic mutations through *Agrobacterium*-mediated transformation.

Tall fescue (2*n* = 6*x* = 42, PPG1G1G2G2) is a widely cultivated cool-season grass used for forage and turf purposes globally. However, its utility is constrained by higher temperatures, particularly during the summer months. Consequently, contemporary breeding efforts are focused on enhancing the grass thermotolerance and extending its green stage to improve its performance under elevated temperature conditions. Khoshhal made one attempt to edit the *SGR* gene in tall fescue by transient expression-based CRISPR-Cas9 system [109]. To establish the CRISPR-Cas9 and Cas12a systems in self-incompatible allohexaploid tall fescue, we firstly identified *FaPDS* as the gene of interest and successfully cloned all three copies of its homologs, namely *FaPDS* -*P*, *FaPDS* -*G1*, and *FaPDS -G2*. To enhance the edit frequency of three homoeoalleles of *FaPDS* at the same time in the T0 plants, we designed sgRNAs or crRNAs that did not have any SNP variations among all three *FaPDS* alleles. By using *Agrobacterium*-mediated calli transformation, we induced specific mutations in all three homoeoalleles of *FaPDS* in tall fescue using CRISPR-Cas9 and Cas12a systems for this purpose. Loss-of-function mutants of *FaPDS* exhibited albino leaves. CRISPR-Cas9 resulted in the creation of 62 mutants, indicating a mutation frequency (MF) ranging from 68% to 83.9%, and CRISPR-Cas12a generated from 13.6% to 61.5% MF [110]. Furthermore, using this CRISPR-Cas9/Cas12a system, we targeted two genes, *FaHSP17.8-CII* and *FaHSP*17.9, and also generated the mutants *fahsp17.8-CII* and *fahsp17.9*, which exhibit high-temperature-sensitive phenotype [16].

Barley is one of the fourth-largest grass species produced worldwide. It belongs to the Poaceae family and is widely used for livestock grass feed [111]. The CRISPR-Cas9 system has been used in barley, but only in a few experiments. Holme *et al*. [112] targeted the barley phytase gene *HvPAPhy_*a promoter and obtained a targeted editing efficiency of 44%. The mature seeds in the targeted mutant had reduced phytase activity and delayed germination. Gasparis *et al*. [113] worked with barley transgenics aiming to discern the effects of *CKX* genes (*CKX1* and *CKX3* involved in the degradation of cytokinin). They generated knock-out mutants for both genes via CRISPR-Cas9 and *Agrobacterium*-mediated transformation of immature embryos. *ckx1* lines reduced CKX enzyme activity in the spikes while *ckx3* lines remained unchanged. Although there were no differences in yield between knock-out mutant lines, the reduction of CKX activity in *ckx1* lines resulted in increased root length, surface area, and root hairs. At the same time, Karunarathne *et al*. [114] worked with the *abnormal cytokinin response 1 repressor 1* (*HvARE1*) gene, which was subjected to genetic engineering (CRISPR-Cas9). One single guide RNA specifically targeting the *HvARE1* gene resulted in the development of 22 T0 plants. Subsequent genotyping identified four T1 lines with mutations causing missense and/or frameshift alterations. Mutant lines were phenotypically higher, had more tillers, and had an improved nitrogen use efficiency. Approximately 1 year ago, without affecting any other important agronomic trait, CRISPR/Cas9-mediated gene editing was used in the *HvGA3ox1* genes of barley to generate barley mutant lines with semi-dwarf height and increased coleoptile length [115].

*E. frumentacea* (Roxb.) Link*.*, commonly known as Japanese millet or Barnyard millet, is a high-quality forage species. Despite its significance, its complex genetic background has led to a scarcity of domestic research [116]. *Leymus chinensis*, commonly known as sheepgrass and belonging to the Poaceae family, is a perennial forage grass renowned for its tolerance to various stresses. An *Agrobacterium*-mediated transformation was optimized, and the *LcTB1* and *GW2* genes were edited efficiently via CRISPR-Cas9 technology [117]. Zoysia grass is a common warm-season forage grass and turfgrass whose widespread use in the northern regions of China is limited due to its short period of green growth [118].

## Challenges and future perspectives for genome editing in grasses

Molecular breeding is an excellent strategy to enhance the breeding capabilities of grasses [119]; however, it faces significant challenges due to the unavailability of desired target loci in grass species. Researchers are more focused on field crops or staple crops and grass species are still can be called “neglected crops” in terms of genomic studies. There is dire need of integrated omics studies to predict genes and correlate the agronomic components of grass species [120]. *In vitro* regeneration of grasses is particularly challenging due to species-specific factors and genetic variations, so there is a need for improved delivery and transformation methods for better efficiency [121]. Although genome editing has been advancing rapidly and being widely used in crop breeding, several obstacles still need to be overcome for its application to grasses. Without complete genome information, cloning genes and evaluating the off-target editing events are challenging when performing genome editing in grasses. For allohexaploid grass species, such as tall fescue, the lack of draft genome sequences further exacerbates the difficulties in conducting molecular and genetic studies. Scientists are actively working to elucidate the functional genomes that control essential traits in grasses and the molecular basis of their interactions with the environment, which significantly limits molecular breeding’s advancements and functional genomics in grasses. Fortunately, with the rapid development of high-throughput sequencing technology, more and more complete maps of grasses whole genomes have been published. These comprehensive genomic maps can be used for the fast identification of candidate genes regulating superior traits and enriching the genetic banks of species, thereby providing genetic resources for molecular breeding by genome editing [122–125]. For example, a CRISPR-Cas9-based genome editing system was successfully established to edit the *PALM1* allele gene and obtain an alfalfa strain without any exogenous transgenic components [110, 126], demonstrating the potential of genome editing to achieve precise genetic improvements. Identification of precise targets of grasses needs to be accelerated which are regulating the phenotypic traits, providing great convenience for future genetic improvement studies of grasses.

Another challenge lies in the development of efficient delivery systems for genome editing in grasses. Nowadays, the central transformation systems used in grasses are *Agrobacterium*-mediated and particle bombardment transformations, both of which depend on the induction of embryonic callus. The leaves could be used as suitable explants for the induction of embryonic callus in many *Leguminosae* types of grass, such as alfalfa. However, most Poaceae grasses, including tall fescue, perennial ryegrass, and so on, must employ seed as the explants to induce embryonic callus. To increase callus induction in Poaceae grasses, sterilized seeds must be sliced longitudinally along the seed groove, dramatically increasing working intensity and cost. Despite these efforts, the effectiveness of the induction of embryonic callus in Poaceae does not match that observed in rice. Additionally, the process usually takes more than 6 months to obtain the regenerated grasses from embryonic calli with a low transformation rate, necessitating extensive effort to screen and identify successfully edited regeneration grasses. Researchers are advancing the development of highly efficient delivery systems for CRISPR-Cas9 that do not require transformation. DNA-free uses CRISPR-Cas9 ribonucleoprotein complexes (RNPs) to transfer into plant cells directly, enabling targeted genes’ the genome editing without transformation [127, 128]. Ma *et al.* [129] completed DNA-free editing in tobacco by using an RNA virus-based vector to systematically deliver CRISPR-Cas9 nucleases to plants and achieve stable inheritance. According to a recent report, a simple cut-dip-budding (CDB) delivery system was established, using *Agrobacterium rhizogene* to infest the shoot-root junction of cut plants, with the upper part of the stem producing transformed roots, which are then segmented and subsequently cultured, and transformed plants grown from roots. The CDB delivery system has been successfully applied to accomplish genetic change in several plant species, including two herbaceous plants, a tuberous root plant, and three woody plants which were used being difficult or impossible to be transformed [11]. A recently modified version of the CDB delivery system was used for random succulent varieties and proved that the CDB delivery system was useful for the production of genetically modified succulent plants [12]. This CDB delivery system allows efficient transformation or gene editing in aseptic conditions without tissue culture. It is shown that the application of the CDB delivery system allows the genetic modification of many plants and has great potential for the genetic modification of grasses [13–15]. Above all, the optimal system should be selected according to the need for genome editing and experimental objectives in applications, while searching for new efficient and low-cost transformation systems and highly efficient tissue culture systems for grasses will help to develop more types of gene-edited grasses in the future.

Additionally, high genome editing efficiency in grasses requires considering the Cas protein type, sgRNA and Cas protein expression levels, etc. New types of Cas with more flexibility in editing activity or shorter PAM sites, such as Cas12a, Cas12b, Cas12i, and Cas12j, as well as CRISPR’s PAMless variants could successfully be useful in crops and offer potential and new options in grasses genome editing. In *Leguminosae* grasses, AtU6 from Arabidopsis was used to drive gRNA expression [71], and OsU6 or OsU3 from rice was employed for gRNA expression in Poaceae grasses. It is highly desirable to establish and develop genome editing techniques with native promoters that shall be deemed to enhance the efficacy of gene editing and greatly facilitate the process of targeted genetic improvement in grasses [130].

All in all, future improvements in genome editing will facilitate wide applications of CRISPR for grass breeding [131]. Although CRISPR-Cas9/Cas12a genome editing technology has edited some targeted genes in grass species, its application is mainly limited to gene knockout, and long and arduous efforts are needed to expand the CRISPR toolkits in grasses [132]. Up-to-date CRISPR toolkits such as CRISPR interference (CRISPRi) systems, CRISPR activation (CRISPRa) systems, BE and engineered plant prime editor (ePPE), for large sequence insertion and replacement, CRISPR libraries in whole plants, multiplex gene editing technology, etc. have been successfully applied in other crops which can be applied to grass species as well [64, 133–135]. Bottlenecks such as editor’s efficiency, transformation efficiency, unavailability of suitable vector and delivery for grass species, ploidy level, gene redundancy, identification of precise genome targets, high-quality genome assemblies [135], and efficient genetic delivery systems with no off-targets, etc. can be introduced into grasses which can revolutionize grass breeding to a progressive potential in the near future.

## Supplementary Material

Web_Material_uhae293
