# Supplementary material for: Genome editing for grass improvement and future agriculture
Source: Hortic Res. 2024 Oct 15;12(2):uhae293. doi: 10.1093/hr/uhae293 (PMC11789526; doi:10.1093/hr/uhae293)
Supplement: Web_Material_uhae293 [file web_material_uhae293.docx]

**Table 1** Genome editing by CRISPR-Cas9/Cas12a and base editors in turf and forage grasses.

| **Familia** | **Species** | **Cas9/**  **Cas12a** | **Targeted gene** | | | **Gene function or Gene Regulated Traits** | **Mutation type** | **Target Sequence*** | **sgRNA efficiency**** | **Delivery method** | **Reference** |
| --- | --- | --- | --- | --- | --- | --- | --- | --- | --- | --- | --- |
|  |  |  | **Name** | **Classification** | |  |  |  |  |  |  |
| ***Leguminosae*** | *Glycine max* | Cas9 | *GS1* | | Metabolism | Glutamine synthase | Deletion | 5′-GAAGTTGGGGAAGAGACACAAGG-3′ | -- | *Agrobacterium*-Mediated Transformation | Michno et al. 2015 |
|  | *Medicago truncatula* | Cas9 | *GUS* | | Reporter | Exogenous marker gene | Deletion | 5′-GTAATTATGCGGGCAACGTCTGG-3′ | -- | *Agrobacterium*-Mediated Transformation | Michno et al. 2015 |
|  | *Medicago truncatula* | Cas9 | *PDS* | | Reporter | Carotenoid biosynthesis | Deletion or base substitution | 5′-CTGGAGGCAAGAGACGTTCTAGG-3′ | 10.35% | *Agrobacterium*-Mediated Transformation | Meng et al. 2017 |
|  | *Medicago truncatula* | Cas9 | *PDS* | | Reporter | Carotenoid biosynthesis | Deletions and insertions | 5′-TGGTCACAAGCCTATATTGCTGG-3′ | 5-75% | *Agrobacterium*-Mediated Transformation | Zhang et al. 2020 |
|  | *Medicago truncatula* | Cas9 | *PDS* | | Reporter | Carotenoid biosynthesis | Deletions and insertions | 5′-GCTGGAGGCAAGAGACGTTCTAGG-3′ | 16.5-70% | *Agrobacterium*-Mediated Transformation | Wolabu et al. 2020 |
|  | *Medicago truncatula* | Cas9 | *NPD* | | Development | Nodule formation | Deletions and insertions | 5′-GATGGAGAATGTGAAGATTAAGG-3′  5′-TTGTACTTAAATGTGACTGGAGG-3′  5′-TGTTGGTGTTGCATGTGAGAAGG-3′  5′-TGGATGATCCTCGTGCTGGGAGG-3′ | -- | *Agrobacterium*-Mediated Transformation | Trujillo et al. 2019 |
|  | *Medicago truncatula* | Cas9 | *CEP* | | Development | Control lateral root & Nodulation | Deletions and insertions | 5′-GAACATTGTGATGGTTCGATGG-3′  5′-GAACATTGTGATGGTTCGATGG-3′  5′-CTACTACTCACTACAACTGAGG-3′  5′-CAAACTTCTATTGCTACGGTGG-3′  5′-TGTTTGATGTCCTACCCCAGGG-3′  5′-AGTCCAGGACATAGCCCTGGGG-3′  5′-GAAGTCTTCAAGACGTGTAGGG-3′  5′-CATCTCCAGGGCATAGTCCAGG-3′  5′-GTCGACGATTTTCGACCAACGG-3′  5′-TGGGAATCTAATGTTGTCGCGG-3′ | -- | *Agrobacterium*-Mediated Transformation | Zhu et al. 2021 |
|  | *Medicago truncatula* | Cas9 | *NCR* | | Development | Symbiotic nitrogen fixation | Deletions and insertions | 5′-GTTGTAATCTTCGAAGATATAGG-3′  5′-CTTGTTTCTTGTTGTAATCTTCG-3′  5′-CTTTTTTGTTCACCTGTTTCTTG-3′ | 30-60%  0%  0-18% | *Agrobacterium*-Mediated Transformation | Güngör et al. 2023 |
|  | *Medicago truncatula* | Cas9 | *CLE35* | | Development | Regulate symbiotic nodule development | Deletions and insertions | 5′-GTTGCTGATGCCACTCACGAGG-3′ | -- | *Agrobacterium*-Mediated Transformation | Lebedeva et al. 2023 |
|  | *Medicago sativa* | Cas9 | *SPL9* | | Development | Squamosa promoter binding protein | Deletions and insertions | 5′-GATGGCGACTCCGTGTTCCCCGG-3′  5′-GCAATCGGTTGGCCTTGGCCGGG-3′ | 2.2% | *Agrobacterium*-Mediated Transformation | Gao et al. 2018 |
|  | *Medicago sativa* | Cas9 | *SGR* | | Metabolism | Regulation of organ senescence & stay green trait | Deletion, insertions and base substitution | 5′-TTCCAAATAGAAGACGGTTATGG-3′  5′-ATTGATGAAGACAAACATCCAGG-3′  5′-GGGATGAAGTTGTGGCGCAGTGG-3′  5′-TGTAACCTCAAAACTCACTTTGG-3′ | 8.4%  8.2%  0%  2.8% | *Agrobacterium*-Mediated Transformation | Wolabu et al. 2020 |
|  | *Medicago sativa* | Cas9 | *GUS* | | Reporter | Exogenous marker gene | Frameshift and non-sense | 5′-GACCGGATGCCGACGCGAAGCGG-3′  5′-ACGACCACGCATTAATGGACTGG-3′ | 48-62% | *Agrobacterium*-Mediated Transformation | Bottero et al. 2021 |
|  | *Medicago sativa* | Cas9 | *SPL8* | | Development | Regulate plant development & Drought tolerance | Deletions and insertions | 5′-GTTCAGGTGGTCTAGACCGACGG-3′  5′-GAGGTTGTGGTTGAGGTGGAGGG-3′  5′-TTGCAGCTGGGTTGACTCAGCGG-3′ | 24.6-72.2%  50.4-57.5%  25.7-29.2% | *Agrobacterium*-Mediated Transformation | Singer et al. 2021 |
|  | *Medicago sativa* | Cas9 | *NP1* | | Development | Male fertility regulator | Deletions and insertions | 5′-GCTACATTGAAGCCGCTAGCGG-3′  5′-CCTTCACAGGTTCTCAAAGAGA-3′ | 96.9% | *Agrobacterium*-Mediated Transformation | Ye et al. 2022 |
|  | *Medicago sativa* | Cas9 | *GA3ox1* | | Development | Gibberellins synthesis | Deletions and insertions | 5′-CCATTCGTTCCCCGGATGGTGT-3′  5′-AAGCCATTCGTTCCCCGGATGG-3′ | -- | *Agrobacterium*-Mediated Transformation | Zheng et al. 2022 |
|  | *Medicago sativa* | Cas9 | *FTa1* | | Metabolism | Key floral integrator & Activator | Deletion, insertions and base substitution | 5′-GTAGGAATCCACTGGCTGTAGGG-3′  5′-AATCAACCCAGAGTGAGTGTTGG-3′  5′-CCCAAGTAACCCCACTTTTAAGG-3′  5′-AGGTTGGTGACTGATATTCCAGG-3′ | 6-8%  25-27%  19%  24-26% | *Agrobacterium*-Mediated Transformation | Wolabu et al. 2023 |
|  | *Medicago sativa* | CBE | *ALS1, ALS2* | | Metabolism | Acetolactate synthase proteins | Base substitution | 5′-CAAGTTCCCCGGAGAATGATCGG-3′  5′-CAGGTTCCCCGGAGAATGATTGG-3′ | -- | *Agrobacterium*-Mediated Transformation | Bottero et al. 2022 |
|  | *Lotus japonicus* | Cas9 | *SYMRK,*  *Lb1/2/3* | | Development | Symbiotic nitrogen fixation | Deletion, insertions and base substitution | 5′-CCTGCAGGTGCGACCGTGGATTG-3′  5′-AGACATGTTCTCCTTTCTAAAGG-3′  5′-ACTCCAAGCCCATGCTGAAAAGG-3′ | 35%  45-65% | *Agrobacterium*-Mediated Transformation | Wang et al. 2016  Wang et al. 2019 |
|  | *Lotus japonicus* | Cas9 | *CZF1, CZF2* | | Development | Regulator of symbiotic nodulation | Deletion | 5′-AGACATGTCAGCTATTGACTTGG-3′  5′- CCTCATACGGAACTGGGCACTGG-3′  5′- CCCGTCTTCAATTTAACTAGCTA-3′  5′- CCGCGCAGATTAACTGAATAGCA-3′ | 0%  39%  0%  46% | *Agrobacterium*-Mediated Transformation | Cai et al. 2018 |
|  | *Lotus japonicus* | Cas9 | *CYP_71_6A_51_* | | Metabolism | Triterpenoid biosynthesis | Deletions and frameshift | 5′-TCTCCCTCTTCTACAAGCACAGG-3′  5′-GAAGATGTGCGAGCAGTACTTGG-3′ | -- | *Agrobacterium*-Mediated Transformation | Suzuki et al. 2019 |
|  | *Lotus japonicus* | Cas9 | *MIR2111-5* | | Development | Nodule formation | Deletion | 5′-ATTGGAAGACGTAATCTGCGTCCCG-3′  5′-ATTGGAAAATGTTGTTCTAAACCTC-3′  5′-ATTGGCCCGAGGACTAGATACAAAA-3′  5′-ATTGGCCTTGTAGCTTGTAGGACTG-3′ | -- | *Agrobacterium*-Mediated Transformation | Okuma et al. 2020 |
|  | *Lotus japonicus* | Cas9 | *α-βCA1-2* | | Metabolism | α-β carbonic anhydrase/CO_2_ fixation | Deletions and insertion | 5′-TTTCTACAGCTGAGGTACAGTGG-3′  5′-AGCGGCTGTGTCCAATGATCAGG-3′  5′-GAAGAATACATCTACACTAAAGG-3′  5′-ATTGGAATATGGTATACAATTGG-3′ | -- | *Agrobacterium*-Mediated Transformation | Wang et al. 2021 |
|  | *Lotus japonicus* | Cas9 | *NIN* | | Development | Root nodule symbiosis & Associated process regulation | Deletion | 5′-TGCGTACATGTCTACGATATCGG-3′  5′-TAAAGAGAGACAGAGAAATAGGG-3′ | -- | *Agrobacterium*-Mediated Transformation | Akamatsu et al. 2022 |
|  | *Trifolium pratense* | Cas9 | *IFS1* | | Metabolism | Isoflavone biosynthesis | Deletion | 5′- GGCGTAACAAGTTGAGACCTTTG -3′ | -- | *Agrobacterium*-Mediated Transformation | Dinkins et al. 2021 |
| ***Poaceae*** | *Brachypodium distachyon* | Cas9 | *PDS* | | Reporter | Carotenoid biosynthesis | Deletions and insertion | 5′-CGTCCAATCCATTCCTCTGCTGG-3′  5′-GATCGTTGCAGAGGTCGACAAGG-3′ | -- | *Agrobacterium*-Mediated Transformation | Hus et al. 2020 |
|  | *Brachypodium distachyon* | Cas9 | *RFS* | | Metabolism | Regulator of flowering & Stress | Deletions and frameshift | 5′-GTGGAAGGCGTCAAGAAATGCG-3′  5′-TGGTACCGTCACCTCGACATGG-3′ | -- | *Agrobacterium*-Mediated Transformation | Ying et al. 2022 |
|  | *Brachypodium distachyon* | Cas9 | *CUL4,*  *LAXA* | | Development | Tillering development | Deletions and insertion | 5′GGCTGTGTTGGGCCAGTTGAGGG-3′  5′-GGCTGTGTTGGGCCAGTTGAGGG-3′  5′-GCCCGCACGCGAGTTCACGTCGG-3′ | -- | *Agrobacterium*-Mediated Transformation | Liu et al. 2021 |
|  | *Festuca arundinacea* | Cas9 | *SGR* | | Metabolism | Regulation of organ senescence & stay green trait |  | 5′-AGCAGCTTCAGCAGCAGCGCCGG-3′ | -- | *Agrobacterium*-Mediated Transformation | Khoshhal 2019 |
|  | *Festuca arundinacea* | Cas9 | *PDS* | | Reporter | Carotenoid biosynthesis | Deletions and insertions | 5′-TCTCTTGGCTCAAGGAATAGAGG-3′  5′-GGATTTACCAAGACCTCCACTGG-3′ | 31%  26% | *Agrobacterium*-Mediated Transformation | Zhang et al. 2021 |
|  | *Festuca arundinacea* | Cas9 | *HSP17.9* | | Stress Response | Heat stress response | Deletions and insertions | 5′-CCCCTTCGACGGCTTCCCCTTCGG-3′  5′- CGGCGCGCGGATCGACTGGAAGG -3′ | -- | *Agrobacterium*-Mediated Transformation | Zhang et al. 2021 |
|  | *Festuca arundinacea* | Cas12a | *PDS* | | Reporter | Carotenoid biosynthesis | Deletions and insertions | 5′-TTTGACGTCTCTTGGCTCAAGGAATAG-3′  5′-TTTCCCAGGATTTACCAAGACCTCCAC-3′ | 26%  22% | *Agrobacterium*-Mediated Transformation | Zhang et al. 2021 |
|  | *Festuca arundinacea* | Cas9 | *HSP17.8-CII* | | Stress Response | Heat stress response | Deletions and frameshift | 5′-CACCTGCTGGACATCCCGGACGG-3′  5′-GACGGCGAGGCCGGCGGAGCCGG-3′ | -- | *Agrobacterium*-Mediated Transformation | Bi et al. 2021 |
|  | *Panicum virgatum* | Cas9 | *tb1a,*  *tb1b/PGM* | | Development | Regulation of tillers production / Phosphoglycerate mutase | Deletion and frameshift | 5′- CCCGATGGACTTACCGCTTTACC -3′  5′-CCCCATGGACTTACCGCTTTACC-3′  5′-CACGGAGCTGGTGGTGGTGCGG-3′ | 95.5%  11%  13.7% | *Agrobacterium*-Mediated Transformation | Liu et al. 2018 |
|  | *Panicum virgatum* | Cas9 | *tb1a,*  *tb1b* | | Development | Regulation of tillers production | Deletion, insertions and base substitution | 5′-CATGGACTTACCGCTTTACCCCA-3′ | 30-94% | *Agrobacterium*-Mediated Transformation | Liu et al. 2020 |
|  | *Lolium perenne* | Cas9 | *DMC1* | | Metabolism | Meiosis regulation | Deletions and insertion | 5′-AGCTTCCACTCCACATGCATGG-3′ | 5.88-11.63% | Particle Bombardment-Mediated Transformation | Zhang et al. 2020 |
|  | *Lolium perenne* | Cas9 | *PDS* | | Reporter | Carotenoid biosynthesis | Deletion | 5′-CTTGAGCTTCAACATAAGCTTGG-3′ | 29% | *Agrobacterium*-Mediated Transformation | Kumar et al. 2022 |
|  | *Leymus chinensis* | Cas9 | *TB1* | | Development | Regulate tiller development | Deletion | 5′-GGTAAAGGGGCAAGTCCATGGGG-3′ | 2.22% | *Agrobacterium*-Mediated Transformation | Lin et al. 2023 |
|  | *Leymus chinensis* | Cas9 | *GW2* | | Development | Regulate tiller development | Deletions and insertion | 5′-CCGGGATGGGGTATTTCTAGAGG-3′ | 5.83% | *Agrobacterium*-Mediated Transformation | Lin et al. 2023 |
|  | *Hordeum vulgare* | Cas9 | *CKX* | | Metabolism | Cytokinin sensing | Deletions and insertion | 5′-GATCACCGCGGCGTCTCCTACGG-3′  5′-CAAGTTCATCCAGAGCCCCATGG-3′ | 12%  8% | *Agrobacterium*-Mediated Transformation | Gasparis et al. 2019 |
|  | *Hordeum vulgare* | Cas9 | *ARE1* | | Metabolism | Cytokinin sensing | Deletion and base substitution | 5′-GAACGATGAGGAAGAGAGCCTGG-3′ | 18-24% | *Agrobacterium*-Mediated Transformation | Karunarathne et al. 2022 |
|  | *Hordeum vulgare* | Cas9 | *GA3ox1* | | Development | Gibberellin (GA) hormone production | Deletions and insertions | 5′-CCGCTCATCGCCTCCTACGTCTC-3′ | 46% | *Agrobacterium*-Mediated Transformation | Cheng et al. 2023 |
|  | *Hordeum vulgare* | Cas9 | *PAPhy_a* | | Development | Contributing to mature grain phytase activity | Deletions and insertions | 5′-GGACGCCTACGTCTCATGCATGG-3′ | 44% | *Agrobacterium*-Mediated Transformation | Holme et al. 2017 |

* Underlined letters are the PAM

**No result in the reference.
